# Chronic kidney disease and HIV in the era of antiretroviral treatment: findings from a 10-year cohort study in a west African setting

**DOI:** 10.1186/s12882-019-1335-9

**Published:** 2019-05-07

**Authors:** Nongodo Firmin Kaboré, Armel Poda, Jacques Zoungrana, Ollo Da, Laura Ciaffi, Aoua Semdé, Issouf Yaméogo, Adrien B. Sawadogo, Eric Delaporte, Nicolas Meda, Sophie Limou, Amandine Cournil

**Affiliations:** 1 Department of Clinical Research, Centre MURAZ, Nongodo Firmin KABORE, Bobo-Dioulasso, BP 808 Burkina Faso; 2 Department of Infectious Diseases, University Hospital Souro Sanou, Bobo-Dioulasso, Burkina Faso; 3 Institut Supérieur des Sciences de la Santé, Université Nazi Boni, Bobo-Dioulasso, Burkina Faso; 4 Biochemistry Department, University Hospital Souro Sanou, Bobo-Dioulasso, Burkina Faso; 5 Unité Mixte Internationale 233, Institut de Recherche pour le Développement, U1175-Inserm, University of Montpellier, Montpellier, France; 6 Department of nephrology, University Hospital Souro Sanou, Bobo-Dioulasso, Burkina Faso; 7 Department of Infectious Diseases, University Hospital of Montpellier, Montpellier, France; 8 Université Ouaga 1 Pr Joseph Ki-Zerbo, Ouagadougou, Burkina Faso; 9 Centre de Recherche en Transplantation et Immunologie (CRTI) UMR1064, Inserm, Université de Nantes, Nantes, France; 10 Institut de Transplantation en Urologie-Néphrologie (ITUN), Nantes University Hospital, Nantes, France; 11 Ecole Centrale de Nantes, Nantes, France; 12 Basic Research Laboratory, NIH/NCI, Frederick National Laboratory, Leidos Biomedical Research, Inc, Frederick, MD USA

**Keywords:** CKD, HIV, Antiretroviral treatment, Burkina Faso, Africa, Epidemiology

## Abstract

**Background:**

It has been reported that people living with HIV in West Africa exhibited the highest risks for chronic kidney disease (CKD) in the world. Here, we aimed at determining the CKD frequency and changes in kidney function during antiretroviral treatment (ART) in a large cohort of HIV-patients followed in Burkina Faso.

**Methods:**

We included ART-naive adults who initiated ART at the Day Care Unit of the Souro Sanou University Hospital between 01/01/2007 and 12/31/2016. We assessed the estimated glomerular filtration rate (eGFR) by serum creatinine using the Modification of Diet in Renal Disease (MDRD) equation. Following the K/DOQI recommendations, CKD was defined as eGFR < 60 ml/min/1.73m^2^ at two consecutive measurements at least 3 months apart. The factors associated with eGFR decline or CKD were identified by mixed linear regression and Cox regression, respectively.

**Results:**

Three thousand, one hundred and thirty-eight patients (72% women) were followed for a median (IQR) of 4.5(2.2–6.9) years. At baseline, median eGFR (IQR) was 110.7(94.4–128.4) ml/min/1.73m^2^ and 93 (3%) patients exhibited eGFR < 60 ml/min/1.73m^2^. The lowest-performing progressions of eGFR during the first year of ART were observed in patients with 40-49 yr. age range (− 8.3[− 11.7;-5.0] ml/min/1.73m^2^, *p* < 0.001), age ≥ 50 yr. (− 6.2[− 10.7;-1.8] ml/min/1.73m^2^, *p* = 0.006) and high blood pressure (HBP) (− 28.4[− 46.9;-9.9] ml/min/1.73m^2^, *p* = 0.003) at ART initiation. Regarding the ART exposure in patients with normal baseline eGFR, zidovudine (AZT) with protease inhibitor (PI) (− 4.7[− 7.7;-1.6] ml/min/1.73m^2^, *p* = 0.002), tenofovir (TDF) + PI (− 13.1[− 17.4;-8.7] ml/min/1.73m^2^, *p* < 0.001), TDF without PI (− 3.2[− 5.0;-1.4] ml/min/1.73m^2^, *p* < 0.001), stavudine (d4T) + PI (− 8.5[− 14.6–2.4] ml/min/1.73m^2^, *p* = 0.006) and d4T without PI (− 5.0[− 7.6–2.4] ml/min/1.73m^2^, *p* < 0.001) were associated with poorer eGFR progression. The prevalence of CKD was 0.5% and the incidence was 1.9 [1.3; 2.7] cases/1000 person-years. The risk of CKD was higher in patients with HBP (4.3[1.8;9.9], *p* = 0.001), 40-49 yr. patients (4.2[1.6;11.2], *p* = 0.004), ≥50 yr. patients (4.5[1.5;14.1], *p* = 0.009) and patients exposed to abacavir (ABC) or didanosine (ddI) based ART (13.1[4.0;42.9], *p* < 0.001).

**Conclusions:**

Our findings do not confirm the high risk of CKD reported in previous studies of West Africans with HIV, but support the recommendations for early initiation of ART and close kidney function monitoring in patients with HBP or aged ≥40 yr.

## Background

Kidney impairments in HIV-infected patients are a major cause of morbidity and mortality [1–3]. In some studies, kidney diseases were reported double the risk of death in HIV-infected patients [4, 5]. On a worldwide scale, the prevalence of chronic kidney disease (CKD) among People living with HIV (PLHIV) is estimated at 6.4%. This prevalence varies across regions, with 7.9% in Africa, 7.1% in North America, 5.7% in Asia and 3.7% in Europe [6]. In the African continent, West Africa has highest rate with a prevalence of 14.6% and Southern Africa is the least affected with a prevalence of 3.2% [6]. The prevalence of HIV-associated nephropathy (HIVAN) decreased with use of antiretroviral treatment (ART) but there remains near a 4-fold increased risk of kidney disease, including CKD, in the PLHIV compared with the general population [7, 8].

Apart from traditional risk factors for CKD such as aging, high blood pressure (HBP) and diabetes, the increased-risk for CKD in PLHIV may be explained by HIV and ART-related factors [9–11]. Several studies have shown high viral load and low CD4 counts as risk factors for CKD or lower glomerular filtration rate (GFR) progression in PLHIV [9, 12–15]. Additionally, antiretroviral drugs are still under tight monitoring regarding their potential renal toxicity. The commonly prescribed Tenofovir Disoproxil Fumarate (TDF) is considered the most nephrotoxic molecule among currently used antiretrovirals [16–19], especially over the short term [19–22]. Beyond TDF, the protease inhibitors (PI), especially lopinavir (LPV) and ritonavir (RTV), are often reported as nephrotoxic [19, 23].

Due to the variety of estimated GFR (eGFR) equations and CKD definitions, it can be challenging to precisely estimate and compare CKD prevalence across published studies. The most accepted definition today has been proposed by the *Kidney Disease Quality Outcome Initiative* (K/DOQI) which defines CKD by the presence of kidney damage or GFR < 60 ml/min/1.73 m^2^ for at least 3 months [24]. Based on this definition, there is very little data on CKD frequency in African cohorts of PLHIV. The objective of this study was to determine the frequency and risk factors for CKD as defined by K/DOQI, as well as the factors predicting the changes in kidney function in PLHIV on ART in sub Saharan Africa.

## Methods

**Fig. 1:**
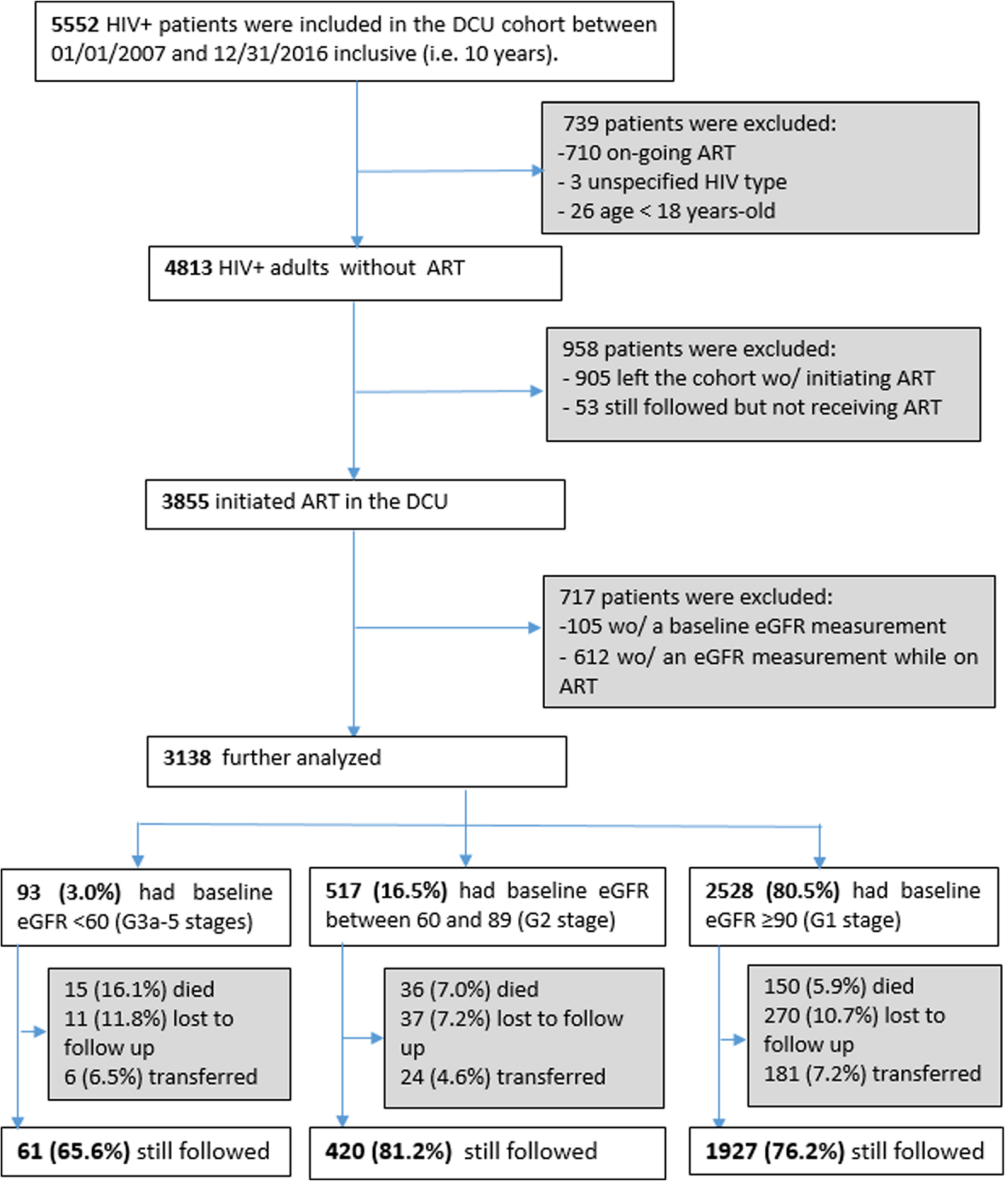
Flowchart of Day Care Unit (DCU) included patients. Wo/: without

We performed the study at the Day Care Unit (DCU) of Sourô Sanou University Hospital in Bobo-Dioulasso, Burkina Faso. The DCU, created in 2005, is part of the Infectious Disease Department and specializes in the care of PLHIV. The ESTHER (Ensemble pour une Solidarité Thérapeutique Hospitalière en Réseau) hospital partnership initiative has supported the implementation of ESOPE, an electronic medical database used to monitor the care of PLHIV. As of 2007, medical records of all patients attending the DCU were entered into the database. Routine clinical follow-up visits were done every 6 months, and all clinical and biological data were recorded in real time by the physician. Patients were included in the present analysis if they were at least 18 yr., had integrated and initiated treatment at the DCU between January 1, 2007 and December 31, 2016, had a serum creatinine measurement at baseline (at treatment initiation or before) and at least another one during the treatment (Fig. 1).

Serum creatinine was estimated by the modified Jaffe’s method on a Konelab 20 (Thermo Electron Oy, Finlande). eGFR was determined using the abbreviated MDRD equation: ((*eGFR = 186 × (Scr(*μmol*) × 0.011312)*^*-1.154*^ *× age(years)*^*-0.203*^ *× 0.742 [if female] × 1.212 [if black]), where Scr is serum creatinine)* [25].

According to the K/DOQI CKD stage definition, analyses were stratified by baseline eGFR level: greater than or equal to 90 ml/min/1.73 m^2^ (G1 stage), between 60 and 89 ml/min/1.73 m^2^ (G2 stage) and less than 60 ml/min/1.73 m^2^ (G3a-5 stages) [24, 26]. For simplicity, we have omitted the eGFR unit (ml/min/1.73m^2^) in the remainder of the manuscript.

Baseline patient characteristics were compared between these three groups using the Kruskal-Wallis test for continuous variables and the Chi-square or Fisher exact test for categorical variables.

Data on proteinuria were not available and CKD was solely defined by eGFR G3a-5 stages (< 60 at two consecutive measurements at least 3 months apart) [24, 26]. Factors associated with CKD were identified using a Cox regression model with time to CKD defined as the time to the first value of eGFR < 60.

Factors associated with changes in kidney function were identified using a mixed linear regression model where the follow-up time after treatment initiation was split into two intervals, i.e. during and after the first year of treatment [27]. The coefficients presented correspond to the differences in slope between the reference category and the other modalities for each variable. Age, sex, body mass index (BMI), CD4 cells count, WHO clinical stage, ART, HBP, and diabetes were tested. Age, sex and the WHO clinical stage were recorded at baseline. BMI, CD4 cells count, ART, HBP, and diabetes were recorded at baseline and during follow-up.

The variables that met the criterion of *P* < 0.25 in a bivariate analysis for association with change in kidney function were retained in the multivariable analysis. All statistical analyses were performed using Stata software (version 14, Stata Corp, College Station, TX, USA). Tests were considered statistically significant for *P* < 0.05.

## Results

### Characteristics of patients at antiretroviral therapy initiation

The baseline characteristics of the patients are shown in Table 1. Seventy-two percent (72%) of the patients were women, and the median serum creatinine (IQR) was 71 (63–83) mmol/μl. The median eGFR (IQR) was 110.7 (94.4–128.4) and 93 (3.0%) patients had a baseline eGFR < 60. By calculating eGFR according to the Cockroft and Gault formula, this number increases to 337 (10.9%) patients. The patients who started ART with a normal baseline eGFR were younger, less immunosuppressed and with less HBP. The three most-prescribed treatment regimens for initial therapy were zidovudine + lamivudine + efavirenz or nevirapine (AZT + 3TC + EFV/NVP) (40.0%), tenofovir + emtricitabine (TDF + FTC) or (TDF + 3TC) + EFV/NVP (31.9%), and stavudine (d4T) + 3TC + EFV/NVP (15.4%). The combination abacavir (ABC) + 3TC + EFV/NVP was the most commonly prescribed protocol for patients initiating ART with eGFR < 60. Patients with impaired kidney function were preferentially prescribed ABC. Median eGFR (IQR) was 45.9 (31.7–67.2) for patients who started ART with therapy containing ABC compared to 109.1 (94.8–125.4), 110.9 (93.5–129.7) and 116.0 (100.0–134.7) respectively for patients who started ART with therapy that contained AZT, TDF and d4T.

**Table 1:** Baseline characteristics at antiretroviral therapy initiation

		eGFR (ml/min/1.73m^2^)				
		≥ 90 *N* = 2528 (80.5%)	60–89 *N* = 517 (16.5%)	< 60 *N* = 93 (3.0%)	*P*-value	Total *N* = 3138
Age (years)		36.2 (30.7–43.2)	42.1 (36.2–48.1)	41.1 (35.8–47.6)	< 0.001	37.3 (31.4–44.5)
Female		1807 (71.5)	390 (75.4)	61 (65.6)	0.072	2258 (72.0)
Age (years)	< 30	546 (21.6)	45 (8.7)	4 (4.3)	< 0.001	595 (19.0)
	[30–40]	1108 (43.8)	168 (32.5)	37 (39.8)		1313 (41.8)
	[40–50]	598 (23.7)	199 (38.5)	34 (36.6)		831 (26.5)
	≥ 50	276 (10.9)	105 (20.3)	18 (19.3)		399 (12.7)
BMI (Kg/m^2^)^a^		20.4 (18.3–23.1)	21.1 (18.6–24.1)	19.5 (17.4–22.2)	< 0.001	20.5 (18.3–23.3)
BMI (Kg/m^2^)^a^	< 18.5	694 (27.5)	123 (24.0)	38 (40.9)	< 0.001	855 (27.3)
	[18.5–25]	1483 (58.8)	287 (55.9)	48 (51.6)		1818 (58.1)
	[25–30]	268 (10.6)	70 (13.7)	6 (6.4)		344 (11.0)
	≥ 30	77 (3.1)	33 (6.4)	1 (1.1)		111 (3.6)
HIV type	1	2371 (93.8)	476 (92.1)	91 (97.8)	0.326	2938 (93.6)
	1 + 2	92 (3.6)	23 (4.4)	1 (1.1)		116 (3.7)
	2	65 (2.6)	18 (3.5)	1 (1.1)		84 (2.7)
WHO stage^a^	1	638 (25.3)	99 (19.4)	8 (8.6)	< 0.001	745 (23.8)
	2	587 (23.3)	111 (21.7)	20 (21.5)		718 (23.0)
	3	1118 (44.3)	252 (49.3)	48 (51.6)		1418 (45.4)
	4	178 (7.1)	49 (9.6)	17 (18.3)		244 (7.8)
CD4 count (cells/μl)^a^		188 (91–288)	169 (93–253)	120 (56–206)	< 0.001	183 (89–279)
CD4 count < 200 cells/μl^a^		1331 (53.4)	295 (57.8)	64 (73.6)	< 0.001	1690 (54.7)
CD4 count < 100 cells/μl^a^		674 (27.1)	139 (27.3)	40 (46.0)	0.001	853 (27.6)
Hemoglobin (g/dL)^a^		10.7 (9.5–12)	10.9 (9.3–12.0)	9.0 (7.6–10.5)	< 0.001	10.7 (9.5–12)
Cholesterol (mmol/ml)^a^		3.5 (2.8–4.1)	3.9 (3.1–4.8)	3.7 (3.0–4.4)	< 0.001	3.5 (2.8–4.2)
Triglyceride (mmol/ml)^a^		1 (0.7–1.3)	1.1 (0.8–1.7)	1.5 (1.1–2.1)	< 0.001	1.0 (0.8–1.4)
ALT (U/L)^a^		18 (12–28)	18 (12–30)	19 (13–33)	0.406	18 (12–29)
Glycemia (mmol/ml)^a^		4.8 (4.4–5.2)	5 (4.6–5.5)	4.9 (4.5–5.4)	< 0.001	4.8 (4.4–5.3)
Diabetes^a^		10 (0.4)	1 (0.2)	1 (1.1)	0.317	12 (0.4)
High blood pressure^a^		187 (7.5)	67 (13.2)	13 (14.0)	< 0.001	267 (8.6)
initial antiretroviral therapies						
AZT/3TC + NNRTI		1031 (40.8)	201 (38.9)	23 (24.7)	< 0.001	1255 (40.0)
AZT/3TC + PI		157 (6.2)	41 (7.9)	4 (4.3)		202 (6.4)
TDF/3TC or TDF/FTC + NNRTI		798 (31.5)	179 (36.6)	25 (26.9)		1002 (31.9)
TDF/3TC or TDF/FTC + PI		68 (2.7)	15 (2.9)	0 (0)		83 (2.6)
TDF/FTC + RAL		1 (0.0)	0 (0)	0 (0)		1 (0.0)
d4T/3TC + NNRTI		399 (15.8)	68 (13.1)	17 (18.3)		484 (15.4)
d4T/3TC + PI		68 (2.7)	6 (1.2)	0 (0)		74 (2.4)
ABC/3TC + NNRTI		4 (0.2)	6 (1.2)	23 (24.7)		33 (1.1)
ABC/3TC + AZT		1 (0.0)	1 (0.2)	0 (0)		2 (0.1)
ABC/3TC or ABC/ddI + PI		1 (0.0)	0 (0)	1 (1.1)		2 (0.1)

Data are median (IQR) or n (%); *eGFR* estimated Glomerular Filtration Rate, *BMI* body mass index, *WHO* World Health Organization, *ALT* alanine aminotransferase, *AZT* Zidovudine, *3TC* Lamivudine, *NNRTI* non-nucleoside reverse transcriptase inhibitor, *TDF* Tenofovir Disoproxil Fumarate, *FTC* emtricitabine, *d4T* Stavudine, *PI* protease inhibitor, *ABC* abacavir, *ddI* didanosine

^a^There were 2 missing data for marital status, 10 missing data for BMI, 13 missing data for WHO stage, 49 missing data for CD4 count, 76 missing data for hemoglobin, 34 missing data for blood pressure, 29 missing data for glycemia, 64 missing data for ALT, 1131 missing data for cholesterol and 1133 missing data for triglyceride

From the 5552 HIV+ patients followed at DCU over the 10-year period, we excluded a total of 2414 patients (Fig. 1). Out of the 958 patients excluded because they had not initiated ART, 660 had at least one measurement of eGFR and the prevalence of eGFR< 60 among them was higher than that of the patients included in the analysis (5.3% vs 3.0%, *p* = 0.002). Out of the 3750 patients who initiated ART and had a baseline measurement of eGFR before starting ART, 612 were excluded because they did not have eGFR measurements during their follow-up on ART (Fig. 1). Those patients were older, more immunosuppressed and had also a prevalence of eGFR < 60 higher than that of patients included in the study (5.6% vs 3.0%, *p* = 0.001).

### Exposures to antiretroviral treatment during follow-up

Patients had a total of 34,874 medical visits, but for statistical analyses, we only retained 34,753 events; the others (121 (0.3%)) being excluded because of nonstandard treatment regimens (bitherapy or monotherapy prescribed during the ANRS 12286 - MOBIDIP study [28].) Of the 34,753 medical visits, patients were exposed to AZT-containing therapy in 56.7% of cases, TDF-containing therapy in 33.3%, and a treatment containing a protease inhibitor in 16.6% of cases including LPVr in 15.9% of cases. The patients who started ART with eGFR < 60 were less exposed to AZT-based therapy (31% vs 57.3%) and more exposed to ABC or didanosine (ddI) based therapy (26.5% vs 2%). Exposures to ART protocols are shown in Table 2.

**Table 2:** Antiretroviral treatments exposure during follow-up

	eGFR (ml/min/1.73m^2^)			
	≥ 90 *N* = 28,289(81.4%)	60–89 *N* = 5645 (16.2%)	< 60 *N* = 819 (2.4%)	Total *N* = 34,753
AZT based cART without PI, n(%)	13,737 (48.6)	2703 (47.9)	205 (25.0)	16,645 (47.9)
AZT based cART + PI, n(%)	2492 (8.8)	521 (9.2)	48 (5.9)	3061 (8.8)
TDF based cART without PI, n(%)	7708 (27.3)	1653 (29.3)	276 (33.7)	9637 (27.7)
TDF based cART + PI, n(%)	1645 (5.8)	284 (5.0)	2 (0.2)	1931 (5.6)
d4T based cART without PI, n(%)	1890 (6.7)	318 (5.6)	71 (8.7)	2279 (6.6)
d4T based cART + PI, n(%)	274 (0.9)	39 (0.7)	0	313 (0.9)
ABC or ddI based cART + PI, n(%)	395 (1.4)	57 (1.3)	16 (2.0)	468 (1.3)
ABC or ddI based cART without PI, n(%)	148 (0.5)	70 (1.0)	201 (24.5)	419 (1.2)

*eGFR* estimated Glomerular Filtration Rate, *cART* combination Antiretroviral Therapy, *AZT* Zidovudine, *PI* protease inhibitor, *TDF* Tenofovir Disoproxil Fumarate, *d4T* Stavudine, *ABC* abacavir

### Estimated glomerular filtration rate changes over time

**Fig. 2:**
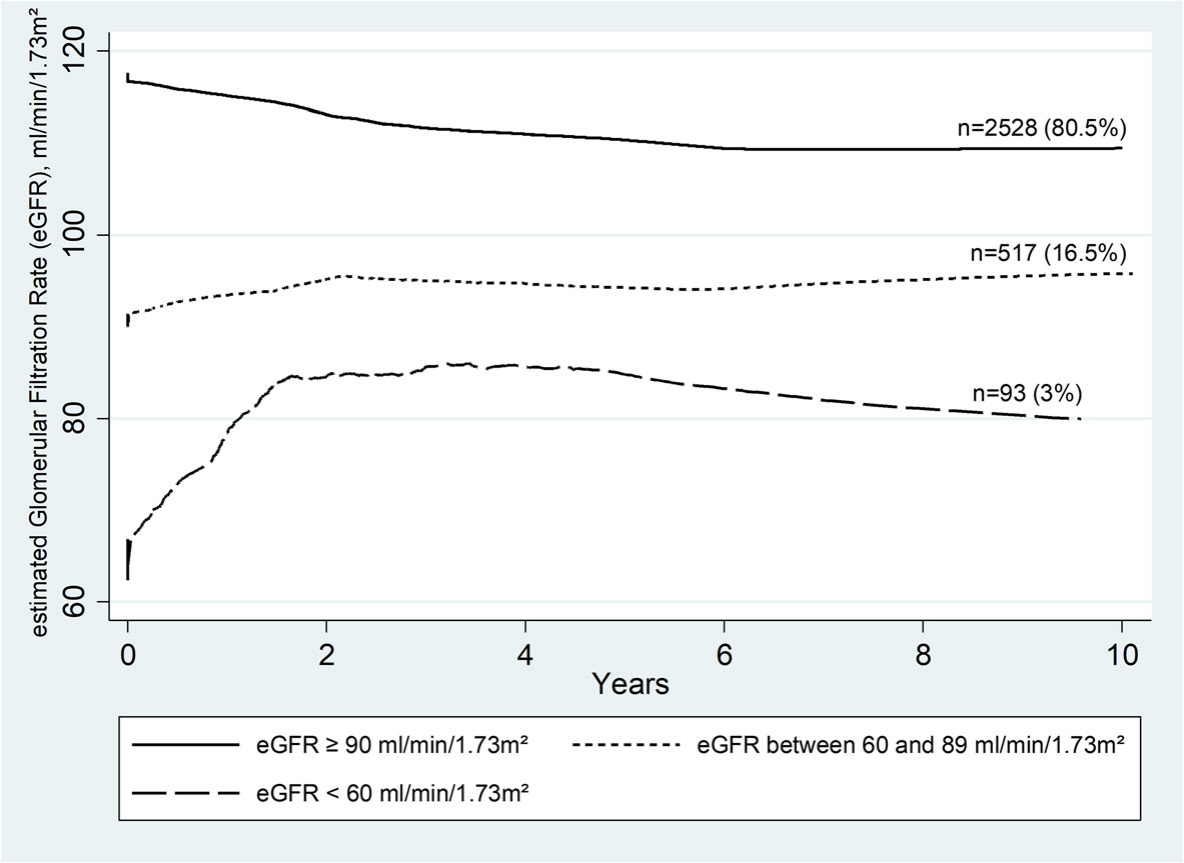
Changes in estimated Glomerular Filtration Rate (eGFR) during antiretroviral therapy, by eGFR baseline. The curves are plotted using the running-mean smoothing

The median duration (IQR) of ART was 4.5 (2.2–6.9) years and 46% of patients had a treatment duration ≥5 years. As illustrated in Fig. 2, the changes in eGFR were more marked at the start of treatment and differed according to the level of baseline eGFR. In patients with normal baseline eGFR (≥90), eGFR decreased on average [95_%_ CI] by 6.8 [6.1; 7.5] in the first year of ART followed by a decrease of 0.4 [0.2; 0.5] per year. For patients with a baseline eGFR between 60 and 89, eGFR increased on average [95_%_ CI] by 11.7 [10.2; 13.2] in the first year, followed by a decrease of 0.5 [0.2; 0.9] per year. In those with a baseline eGFR < 60, eGFR increased on average [95_%_ CI] by 35.4 [30.3; 40.6] in the first year, followed by a decrease of 2.2 [0.3; 4.1] per year.

### Changes in estimated glomerular filtration rate over time

Regardless of the level of eGFR at ART initiation, the major eGFR changes appeared during the first year of treatment. Factors influencing changes in eGFR (i.e., had significant interactions with time) are shown in Table 3.

**Table 3:** Predictors of changes in estimated glomerular filtration rate

		eGFR^a^ (ml/min/1.73m^2^/year)	CI_95%_	*p*-value	eGFR^a^ (ml/min/1.73m^2^/year)	CI_95%_	*p*-value
		During first year			After first year		
Baseline eGFR ≥ 90 ml/min/1.73m^2^, *N* = 2528							
Baseline age, years	Ref (< 40)	–	–	–	–	–	–
	40–49	1.7	[− 0.1;3.5]	0.060	0.2	[− 0.2;0.6]	0.242
	≥ 50	−0.7	[− 3.2;1.8]	0.590	0.6	[−0.2;1.0]	0.197
Baseline BMI ≥ 25 Kg/m^2^		0.3	[− 1.9;2.5]	0.787	0.5	[0.0;1.0]	0.050
Baseline High Blood Pressure		0.3	[−2.5;3.1]	0.829	0.1	[−0.5;0.7]	0.738
WHO stage ≥3		0.4	[−1.1;2.0]	0.591	−0.2	[−0.5;0.1]	0.236
Baseline CD4 count < 100 cells/μl		−2.7	[−4.4;-1.0]	0.002	− 0.5	[− 0.8;-0.1]	0.015
cART exposure	Ref(AZT without PI)	–	–	–	–	–	–
	AZT + PI	−4.7	[−7.7;-1.6]	0.002	0.1	[−0.5;0.6]	0.856
	TDF without PI	−3.2	[−5.0;-1.4]	< 0.001	−0.2	[− 0.6;0.2]	0.326
	TDF + PI	−13.1	[− 17.4;-8.7]	< 0.001	− 0.1	[−0.7;0.6]	0.838
	d4T without PI	−5.0	[−7.6;-2.4]	< 0.001	2.7	[1.0;4.4]	0.002
	d4T + PI	−8.5	[− 14.6;-2.4]	0.006	3.4	[−1.4;8.2]	0.171
	ABC/ddI	0.2	[−12.8;13.1]	0.981	0.1	[−1.0;1.2]	0.884
Baseline eGFR between 60 and 89 ml/min/1.73m^2^, *N* = 517							
Baseline age, years	Ref (< 40)	–	–	–	–	–	–
	40–49	−8.3	[−11.7;-5.0]	< 0.001	0.9	[0.1;1.6]	0.028
	≥ 50	−6.2	[−10.7;-1.8]	0.006	0.7	[−0.4;1.9]	0.193
Baseline BMI ≥ 25 Kg/m^2^		−2.2	[−6.1;1.7]	0.273	0.8	[−0.1;1.7]	0.076
Baseline High Blood Pressure		−2.7	[−7.4;2.0]	0.258	0.5	[−0.6;1.6]	0.362
WHO stage ≥3		2.5	[− 0.7;5.7]	0.121	−0.2	[−0.9;0.6]	0.652
Baseline CD4 count < 100 cells/μl		1.1	[−2.5;4.6]	0.549	−1.1	[−2.0;-0.3]	0.009
cART exposure	Ref(AZT without PI)	–	–	–	–	–	–
	AZT + PI	0.2	[−5.4;5.8]	0.947	− 0.7	[−1.9;0.5]	0.239
	TDF without PI	1.0	[−2.7;4.7]	0.590	−0.2	[−1.0;0.6]	0.631
	TDF + PI	−7.2	[−15.8;1.6]	0.111	−1.1	[− 2.6;0.5]	0.167
	d4T without PI	6.3	[0.5;12.1]	0.034	−1.3	[−6.6;3.6]	0.601
	d4T + PI	−0.3	[−16.7;16.1]	0.974	−20.7	[− 31.8;-9.5]	< 0.001
	ABC/ddI	−6.6	[−19.5;6.2]	0.313	2.4	[0.4;4.5]	0.018
Baseline eGFR < 60 ml/min/1.73m^2^, *N* = 93							
Baseline age, years	Ref (< 40)	–	–	–	–	–	–
	40–49	5.4	[−7.2;18.0]	0.398	2.5	[− 3.7;8.6]	0.435
	≥ 50	−5.6	[−22.1;10.9]	0.506	−2.4	[−10.3;5.6]	0.564
Baseline BMI ≥ 25 Kg/m^2^		−14.8	[−41.2;11.6]	0.272	3.3	[−8.5;15.1]	0.581
Baseline High Blood Pressure		−28.4	[−46.9;-9.9]	0.003	−0.7	[−9.4;8.1]	0.879
WHO stage ≥3		6.4	[−6.7;19.5]	0.336	3.6	[−2.2;9.5]	0.223
Baseline CD4 count < 100 cells/μl		7.9	[−3.6;19.3]	0.179	4.8	[−0.8;10.4]	0.095
cART exposure^b^	Ref(AZT without PI)	–	–	–	–	–	–
	AZT + PI	9.3	[−20.3;38.9]	0.537	10.0	[− 0.3;20.3]	0.058
	TDF without PI	−18.9	[−33.9;-3.8]	0.014	1.4	[−2.8;5.6]	0.520
	d4T without PI	−22.2	[−41.5;-2.9]	0.024	−26.1	[−51.6;-0.5]	0.045
	ABC/ddI	−19.6	[−36.2;-3.0]	0.020	2.5	[−3.6;8.5]	0.425

*eGFR* estimated Glomerular Filtration Rate, *CI* confidence interval, *BMI* body mass index, *WHO* World Health Organization, *cART* combination Antiretroviral Therapy, *AZT* Zidovudine, *PI* protease inhibitor, *TDF* Tenofovir Disoproxil Fumarate, *d4T* Stavudine, *ABC* abacavir, *ddI* didanosine

^a^The coefficient gives the difference of eGFR change in each period between the modality of the variable presented in the table and the reference category. A positive coefficient indicates a more favorable evolution whereas a negative coefficient indicates a less favorable evolution

^b^2 patients only were exposed to TDF + IP containing therapy

In patients who initiated ART with normal eGFR, the predictive factors for greater eGFR decline in the first year were exposure to AZT + PI (*p* = 0.002), TDF without PI (*p* < 0.001), TDF + PI (*p* < 0.001), d4T without PI (*p* < 0.001) or d4T + PI (*p* = 0.006). Low CD4 cell counts (< 100 cells/μl) at the initiation of ART were associated with greater decline of eGFR during the first year of ART (*p* = 0.002) and beyond (*p* = 0.015).

For patients who had a baseline eGFR between 60 and 89, the predictive factors for poor eGFR progression in the first year of treatment were age: 40-49 yr. (*p* < 0.001) and ≥ 50 yr. (*p* = 0.006). Predictors of poor eGFR progression beyond the first year of treatment were low CD4 cell counts (< 100 cells/μl) at ART initiation (*p* = 0.009), and exposure to treatment containing both d4T and a PI (*p* < 0.001).

In patients who started ART with eGFR < 60, the predictive factors for poor eGFR progression in the first year were HBP (*p* = 0.003) at ART initiation and exposure to TDF-containing ART (*p* = 0.014) or ART containing ABC, ddI or both (*p* = 0.020). Exposure to d4T-containing ART was associated with poor eGFR progression in the first year of ART (*p* = 0.024) and beyond (*p* = 0.045).

### Prevalence, incidence and risk factors for chronic kidney disease (CKD)

Among the 3138 patients, we observed 14 cases of CKD (eGFR < 60 at ART initiation and confirmed at least 3 months). The analyses therefore focused on 3124 patients who contributed to 14,318 person-years of follow-up. A total of 27 incident cases of CKD were recorded, namely 1.9 [1.3; 2.7] cases per 1000 person-years. In a multivariable analysis, the risk of CKD was 4.3 [1.8;9.9] times higher in patients with HBP (*p* = 0.001). Compared with younger patients (40 yr), the risk of CKD was respectively 4.2 [1.6;11.2] times (*p* = 0.004) and 4.5 [1.5;14.1] times (*p* = 0.009) higher in the 40-49 yr. and ≥ 50 yr. age groups. Compared to patients who were on AZT-containing therapy without PI, those who were exposed to therapy containing ABC, ddI or both were 13.1 [4.0;42.9] times (*p* < 0.001) more likely to develop CKD. (Table 4).

**Table 4:** Risk factors for chronic kidney disease during antiretroviral therapy

		HR	CI_95%_	*p*-value
Baseline age (years)	Ref (< 40)	–	–	–
	40–49	4.2	[1.6;11.2]	0.004
	≥50	4.5	[1.5;14.1]	0.009
Baseline high blood pressure		4.3	[1.8;9.9]	0.001
cART exposure	AZT without PI	–	–	–
	AZT + PI	0.5	[0.1;4.2]	0.542
	TDF without PI	1.6	[0.6;4.2]	0.347
	TDF + PI	2.3	[0.5;10.4]	0.300
	d4T without PI	2.0	[0.4;9.8]	0.393
	d4T + PI	6.7	[0.8;55.9]	0.079
	ABC/ddI	13.1	[4.0;42.9]	< 0.001

*HR* hazard ratio, *CI* confidence interval, *cART* combination Antiretroviral Therapy, *AZT* Zidovudine, *PI* protease inhibitor, *TDF* Tenofovir Disoproxil Fumarate, *d4T* Stavudine, *ABC* abacavir, *ddI* didanosine

## Discussion

In our cohort of patients from Burkina Faso, followed for 10 years after therapy initiation, mean eGFR improved during the first year of treatment in patients with kidney impairment (eGFR < 90) at ART initiation. Baseline predictors for poor kidney function progression after initiation of ART were older age (≥40 yr), HBP and low CD4 counts (< 100 cells/μl). Exposure to treatments containing TDF or d4T, especially when associated with PI was also a predictor for poor kidney function evolution. The incidence of CKD was low (1.9 per 1000 patient-years) in our cohort as was CKD prevalence (0.5%). Patients over 40 and those with HBP were more likely to progress to CKD during follow-up. Patients who received ABC for their kidney impairment were also more likely to progress to CKD.

Consistently with the findings of previous reports in cohorts of adults initiating ART, we found that baseline eGFR was a strong predictor of subsequent changes and eGFR increased in individuals with impaired kidney function at treatment initiation [13, 29, 30]. The lower the baseline eGFR, the higher the improvement was, especially in the first year following ART initiation.

Of the three equations commonly used to estimate eGFR, the Cockcroft and Gault equation is the least accurate with a lower eGFR than those estimated by the MDRD and Chronic Kidney Disease Epidemiology Collaboration (CKD-EPI) equations [31]. Therefore, we will not discuss here prevalences based on the Cockcroft and Gault equation.

Based on the confirmed CKD as defined (stage 3a-5 on 2 measurements), two African studies reported different prevalences. One in Burundi with a prevalence close to what we observed (1.7% = 5/300) [32] and the other in Nigeria, with a much higher prevalence (9.3%) [33]. In the Burundian study, nearly 30% of patients had not yet initiated ART and the prospective design allowed the second measurement of eGFR to be performed within 3 months. As for the Nigerian study, which is also based on a database analysis like ours, it was characterized by a very high prevalence of HBP compared to that observed in our cohort (45.7% vs 8.6%). In a cohort study of PLHIV in the United Kingdom (UK), the prevalence of CKD was 4.3% among people from West Africa. This prevalence derived from a population predominantly of Nigerian and Ghanaian origin (73%) and was higher than that observed in our cohort [34].

Based on the unconfirmed CKD defined by an eGFR < 60 on a single measurement, we find more studies in Africa. In our study, the prevalence of baseline eGFR< 60 is 3%. This prevalence is comparable to the prevalence found in a study that included Zimbabwe and Uganda patients (3.1%) [35] and in another Rwanda study (2.7%) [36]. Within West Africa, the prevalence observed in our study is lower than the ones reported in Ghana (13.7%) [37] and Nigeria (16.3 to 24%) [38, 39] even though a recent study reported a prevalence of 3% for eGFR < 60 in ART-naïve HIV-infected patients in Nigeria [40]. In West Africa, most studies on the prevalence of CKD among PLHIV have been performed in Ghana and Nigeria. Most of these studies report higher prevalence rates than those observed in our study [6, 37–39]. In the general population, the reported prevalences fluctuate with 1.8% in Côte d’Ivoire [41], 1.6% in Ghana [31] and 12.3% in Nigeria [42]. Additional studies in PLHIV in West African, including countries other than Ghana and Nigeria, are called for to assess CKD prevalence in this region.

Studies in Africa on CKD incidence among PLHIV are based on unconfirmed CKD [13, 43]. To our knowledge, our study is the first on the continent to assess the incidence of CKD by two consecutive measures and we report here a MDRD-based CKD incidence of 1.9 [1.3;2.7] per 1000 person-years. Cohort studies performed in high income countries with a similar CKD definition, reported higher incidence in PLHIV with similar age range. In Europe, the MDRD-based CKD incidence was 3.9–16.1 cases per 1000 person-years in a French retrospective cohort study [14] and the CKD-EPI-based CKD incidence was 7.9[6.0–9.9] in participants from West Africa followed up in an UK cohort [34]. In two USA cohort studies including > 1/3 African-Americans, the CKD-EPI-based CKD incidences were 10.1 [8.3–12.3] and 5 [4.2;6.0] per 1000 person-years [9, 15]. People with baseline impaired kidney function and poor clinical condition are at higher risk for mortality [1, 37, 44]. Baseline median CD4 count in our cohort was lower than in American, UK and French cohorts. Low baseline CD4 count and delay in CKD diagnosis (biological assessment only every 6 months) may have contributed to an increased mortality in our most fragile kidney patients, before eventual confirmation of the chronicity of their kidney disease.

In accordance with our results, most studies found that older age and HBP are predictors of poor kidney function progression and of CKD [9, 11, 13–15, 29, 43]. Aging leads to a physiological decline in eGFR of about 8 ml/min/1.73 per decade after 40 years [45]. Over time, HBP damages the kidneys’ blood vessels and leads to CKD [46]. History of kidney disease, hepatitis C and diabetes were also identified as predictors of CKD in some studies but we were unable to test these factors because this information not available in our cohort [9, 11, 14, 15]. Some reports also emphasized low CD4 cell count as a predictor of CKD, but in our analysis, CD4 count was associated with a decline in kidney function only in the group initiating ART with normal eGFR values [9, 11, 13–15]. Regarding the impact of antiretroviral drugs, we have confirmed the nephrotoxic effect of TDF [14–19, 47] and have reported that this nephrotoxicity was increased when the TDF was associated with PI, which suggests a potentiating effect of PI [48]. AZT compared to other nucleoside reverse transcriptase inhibitors (NRTI), showed better kidney safety in our study. Several studies have suggested a possible nephrotoxicity of d4T and ddI, while ABC seems to be safe for the kidney [14, 49, 50]. For this reason, in our cohort, ABC was preferentially reserved for patients with kidney insufficiency and this could explain its association with kidney impairments in our study.

### Limitations of study

In this study, we cannot rule out the contribution of a selection bias to explain the low prevalence and incidence of CKD in the cohort. Indeed, we showed that patients who were not included in the analysis because they died or were lost to follow-up before initiating ART or before having eGFR follow-up data while on ART, were more likely to have eGFR< 60 at baseline. This observation supports a selection or survival bias that would lead to underestimation of CKD prevalence and incidence in the population. This selection bias may have had a higher impact in this study compared to other studies because of the retrospective design of the study with less frequent biological monitoring. Another limitation of our study is the unavailability of certain data, including proteinuria and viral load at initiation of antiretroviral therapy. Similarly, information on concomitant medications including possible nephrotoxic drugs (nonsteroidal anti-inflammatory drugs, traditional medicines, etc.), hepatitis C or a history of kidney disease were not available. This may have introduced confounding biases into our analyses.

## Conclusions

Our study reports a CKD prevalence of 0.5% and an incidence of 1.9 per 1000 person-years in a cohort of PLVIH from Burkina Faso. These numbers are lower than previously reported. The retrospective design of our study with less frequent measurements of eGFR and the process of selecting our study population might have underestimated the CKD frequency in our cohort. HBP and age ≥ 40 yr. at ART initiation are both risk factors for CKD and predictors for poor eGFR progression during ART. Patients who started ART with kidney impairments had their kidney function improved but never recovered to an eGFR level similar to those who started ART with normal kidney function. In patients who started ART with a normal kidney function, exposure to ART containing both TDF and PI induced the most important loss of eGFR (− 13.1 [− 17.4;-8.7]) during the first year of treatment.

In conclusion, our results support the recommendations for early ART initiation before the onset of kidney impairment, which is concomitant with immunosuppression. We also suggest close kidney function monitoring in PLHIV with HBP and those aged ≥40 yr. The use of treatment containing both TDF and PI should be cautious in patients with renal risk. Finally, a prospective cohort study will be necessary to better estimate the prevalence and incidence of CKD among PLHIV in Africa.

## Abbreviations

3TC: Lamivudine
ABC: Abacavir
ALT: Alanine aminotransferase
ART: Antiretroviral treatment
AZT: Zidovudine
BMI: Body Mass Index
cART: combination Antiretroviral Therapy
CI: Confidence Interval
CKD: Chronic kidney disease
CKD-EPI: Chronic Kidney Disease Epidemiology Collaboration
d4T: Stavudine
DCU: Day Care Unit
ddI: didanosine
eGFR: estimated glomerular filtration rate
ESTHER: Ensemble pour une Solidarité Thérapeutique Hospitalière en Réseau
FTC: Emtricitabine
HBP: High blood pressure
HIVAN: HIV-associated nephropathy
IQR: Inter quartile range
K/DOQI: Kidney Disease Quality Outcome Initiative
MDRD: Modification of Diet in Renal Disease
NNRTI: Non-Nucleoside Reverse Transcriptase Inhibitor
PI: Protease Inhibitor
PLHIV: People living with HIV
RTV: Ritonavir
SCr: Serum creatinine
TDF: Tenofovir Disoproxil Fumarate
UK: United Kingdom
WHO: World Health Organization
